# Piacentinu Ennese PDO Cheese as Reservoir of Promising Probiotic Bacteria

**DOI:** 10.3390/microorganisms7080254

**Published:** 2019-08-12

**Authors:** Alessandra Pino, Nunziatina Russo, Koenraad Van Hoorde, Maria De Angelis, Giuseppe Sferrazzo, Cinzia Lucia Randazzo, Cinzia Caggia

**Affiliations:** 1 Department of Agricultural, Food and Environment, University of Catania, 95123 Catania, Italy; 2 Department of Biotechnology, Laboratory of Brewing Science and Technology, Faculty of Bioscience Engineering, Ghent University, 9000 Ghent, Belgium; 3 Department of Soil, Plant and Food Science, University of Bari Aldo Moro, 70121 Bari, Italy; 4 Department of Drug Sciences, Section of Biochemistry, University of Catania, 95125 Catania, Italy

**Keywords:** lactic acid bacteria, traditional cheese, safety assessment, functional properties

## Abstract

Piacentinu Ennese is a protected designation of origin (PDO) cheese produced in the surrounding area of Enna (Sicily, Italy), using raw ewe’s milk without the addition of any starter cultures. In the present study, the *Lactobacillus* population of Piacentinu Ennese PDO cheese was in vitro screened in order to select promising probiotic strains to be further used in humans. One hundred and sixty-nine lactic acid bacteria (LAB) were isolated from 90 days ripened cheeses and identified by Rep-PCR genomic fingerprinting, using the (GTG)_5_-primer, and by MALDI-TOF MS. One hundred and thirteen (113) isolates belonging to QPS-list species were characterized for both safety and functional properties. All tested isolates were considered safe because none showed either gelatinase, DNase, mucinase, or hemolytic activity. Tolerance to lysozyme, bile salts, and acidic conditions, along with ability to survive under simulated gastrointestinal digestion, were observed. In addition, based on antimicrobial activity against pathogens, cell surface characteristics, Caco-2 adhesion abilities, and anti-inflammatory potential, it was possible to confirm the strain-dependent functional aptitude, suggesting that Piacentinu Ennese PDO cheese may be considered a precious source of probiotic candidates.

## 1. Introduction

In recent years, the increasing consumer demand for foods with health-promoting properties has stimulated both scientific research and the food industry to innovate and develop new products [1]. This trend is in accordance with the hypothesis that a balanced diet, beyond satisfying nutritional needs, may help to maintain a healthy status throughout life, preventing not only gastrointestinal disorders but also chronic diseases (e.g., cardiovascular dysfunctions, cancer, osteoporosis) [2,3,4,5,6]. In this context, functional foods, defined as processed foods containing ingredients that aid specific body functions in addition to being nutritious [7], can be considered as health fellows. Functional foods include products with cholesterol-lowering properties, fortified foods (with vitamins, minerals, bioactive peptides, or ω3-polyunsaturated fatty acids) or deprived foods (of fat, sugar, or salt) as well as those with added probiotic bacteria [8,9]. Probiotics, recognized as live bacteria that when administered in adequate amount confer health benefits to the host [10], are widely used in food and feed as a non-pharmacological approach to health management [11]. Although there is a reasonable number of well-characterized probiotic strains [12,13,14], the isolation and characterization of new ones is still desirable [15]. In fact, since probiotic properties and health benefits are generally “strain-specific” and may not be extended to other strains of the same genus or species [16,17,18,19], it is of high relevance to screen for new strains with health benefits [20]. 

Generally, strains used as probiotics are of human origin, but this is not a requirement. In fact, the FAO/WHO definition of probiotic does not mention the human origin of the bacterial strain as criteria for the selection of probiotics and, instead, it is based on the type of effect caused [10,15,19]. According to that, different food matrices have been used as probiotic isolation source. In particular, foods containing probiotic bacteria are strongly linked to fermented dairy products, such as cheese, which is considered both a source and a good carrier of probiotics [21,22]. Cheese is characterized by optimal pH values (ranging from 4.8 to 5.6), good buffering capacity, and high fat content, which protects probiotics during the passage through the gastrointestinal tract (GIT) [23]. In addition, cheese contains essential nutrients, such as biologically valuable protein, conjugated linoleic acid (CLA), minerals, such as calcium, phosphorus, and magnesium, as well as folic acid, vitamin B6, and vitamin B12. Several studies have already confirmed the high functional value of cheese, including its ability to support the growth/survival of probiotic strains [24,25,26,27]. Among traditional cheeses, Piacentinu Ennese (PE) is a pressed protected designation of origin (PDO) cheese produced using raw ewe’s milk without the addition of any starter culture. The PE cheese is typically cylindrical, with a weight ranging from about 3.5 to 4.5 kg and is generally consumed as semi-hard (after 2–4 months of ripening) or hard (over 4 months of ripening) cheese type. During cheese-making, saffron (*Crocus sativus*), which confers a bright yellow color, and whole peppercorns are added, contributing to the typical spicy aroma and flavor of the final product [28,29]. 

Based on our knowledge, this is the first study aimed to characterize the *Lactobacillus* population of PE cheese from the functional point of view. The primary outcome of the present study was to select at least five strains with promising probiotic properties. According to that, lactobacilli, isolated from 90 days-ripened PE cheese samples, were in vitro screened based on traits qualifying them as potential probiotics suitable for further in vivo studies.

## 2. Materials and Methods 

### 2.1. Cheese Samples and LAB Isolation

The PE cheese samples, manufactured following traditional practices, were provided by three different farms located in the surrounding area of Enna (Sicily, Italy). In detail, nine ripened cheese samples, obtained from three independent batches, were collected and transferred to the Laboratory of Microbiology (Department of Agricultural, Food, and Environment, University of Catania) under refrigerated conditions (4 ± 2 °C). Each sample (25 g) was from duplicate cores taken from one cheese. Cheese samples were subjected to lactic acid bacteria (LAB) isolation as previously reported [30], using de Man, Rogosa and Sharpe (MRS) (Oxoid, Milan, Italy) agar medium, adjusted to pH 5.4, incubated at 32 °C for 72 h under microaerophilic conditions. For each cheese sample, 30% of the total number of colonies recovered on MRS agar plates at highest dilutions were randomly selected and purified before storing in liquid culture using 20% glycerol at −80 °C. Overall, 225 isolates were obtained and 169 of them were presumptively considered LAB based on microscopic examination, positive Gram reaction, absence of catalase activity and spore formation and non-motility.

### 2.2. LAB Identification

Total genomic DNA was extracted from the LAB isolates following the protocol described by Pino et al. [31]. DNA concentration and quality were checked by Fluorometer Qubit (Invitrogen, Carlsbad, 278 CA, USA). LAB identification was carried out by Rep-PCR genomic fingerprinting, using the (GTG)_5_-primer, and MALDI-TOF MS (Bruker Daltonics, Kontich, Belgium) as previously reported by Pino and co-workers [31] and Russo and co-workers [32], respectively.

### 2.3. Reference Strains and Culture Conditions

*Streptococcus pyogenes* ATCC 19615 and *Streptococcus pneumoniae* ATCC 6303, used as positive controls for hemolytic activity, were cultured on brain heart infusion (BHI, Becton Dickinson GmbH, Germany) at 37 °C under 5% of CO_2_, while *Listeria monocytogenes* DSM 12464 and *Salmonella enterica* serovar *typhimurium* ATCC 14028 were reactivated on the same medium at 30 °C under aerobic conditions. *Escherichia coli* 555 and *Staphylococcus aureus* ATCC 6538 were cultured on trypticase soy broth medium (Oxoid, Milan, Italy) at 37 °C, under aerobic conditions.

### 2.4. Safety Assessment of LAB Isolates

#### 2.4.1. DNAse, Gelatinase, Hemolytic and Mucin Degradation Activities 

The LAB strains were evaluated for DNAse and gelatinase activities, according to Lavilla-Lerma and co-workers [33]. Mucin degradation ability was determined according to Muñoz-Atienza et al. [34]. Hemolytic activity was evaluated on blood agar plates containing sheep blood (Biolife, Milan, Italy) by streaking overnight cell cultures. After incubation at 37 °C for 24–48 h, the hemolytic activity was visually detected and distinguished as β-hemolysis, α-hemolysis, or γ-hemolysis based on the appearance of a clear zone, green halo or no zones around colonies, respectively. *S. pyogenes* ATCC 19615 and *S. pneumoniae* ATCC 6303 were used as positive controls for β-hemolysis and α-hemolysis, respectively. All analyses were performed in triplicate. 

#### 2.4.2. Biogenic Amine Production

The ability of the LAB strains to produce biogenic amines (BA) was tested according to Bover-Cid and Holzapfel [35], using tyrosine (freebase), histidine monohydrochloride, ornithine monohydrochloride and lysine monohydrochloride (all purchased from Sigma, St. Louis, MO, USA) as precursor amino acids. The strains were subcultured twice in MRS broth containing 0.1% of each precursor amino acid and 0.005% of pyridoxal-5-phosphate. Overnight cell cultures were streaked on MRS agar containing 0.006% of bromocresol purple, 0.005% piridoxal-5-phosphate and 1% of each tested amino acid. The pH of the medium was adjusted to 5.3 by adding 1 M HCl. After incubation at 37 °C for 4 days, plates were evaluated for the presence of purple color in the surrounding colonies. Plates without amino acids were used as controls. Analyses were carried out in triplicate. 

#### 2.4.3. Antibiotic Susceptibility and MIC Determination

The LAB strains were considered resistant or sensitive to each tested antibiotic (ampicillin, vancomycin, gentamycin, kanamycin, streptomycin, erythromycin, clindamycin, tetracycline, chloramphenicol) according to breakpoints proposed by the European Food Safety Authority (EFSA) [36]. The minimum inhibitory concentration (MIC) was determined by the Etest^®^ method (BioMérieux, Marcy l’Etoile, France), using the LAB susceptibility test medium (LSM) agar, consisting of a mixture of Iso-Sensitest agar (Oxoid) (90%) and MRS agar (10%) (pH 6.7), as recommended by ISO 10932/IDF 223 [37]. In detail, individual colonies, grown on LSM agar for 24 h at 37 °C, were suspended in a 0.85% NaCl solution to reach a McFarland standard value of 0.5, then were swabbed on LSM agar and dried before applying the Etest^®^ strips. After incubation at 37 °C for 48 h, MIC values were determined following the manufacturer’s instructions. Strains showing MICs lower or higher than the EFSA’s breakpoints were considered as sensitive or resistant, respectively [36]. All analyses were performed in triplicate. 

### 2.5. Lysozyme, Acidic and Bile Salts Tolerances

Lysozyme tolerance was evaluated according to Caggia and co-workers [24]. Aliquots, collected at 0, 30, and 120 min, were subjected to viable bacteria cells count (log CFU/mL) on MRS agar plates. Bacterial cells, suspended in a sterile electrolyte solution without the addition of lysozyme, were used as control. 

Acidic tolerance was tested on MRS broth adjusted to pH 2.0 and 3.0 with 1M HCl. MRS broth at pH 6.2 was used as control. In detail, bacterial suspension (9 log CFU/mL) was inoculated into acidified medium, and aliquots were taken at 0, 2, and 4 h of incubation at 37 °C. Tolerance to the tested acidic conditions was evaluated by viable cells count on MRS agar plates, incubated at 37 °C for 48 h, and expressed as survival rate percentage (SR %), based on the initial and the final number of viable cells. Bile salts tolerance was evaluated using MRS broth with 0.5% and 1.0% of bovine bile salts (Oxgall; St. Louis, MO, USA, Sigma-Aldrich). MRS broth without bovine bile salts was used as control. LABs were inoculated at final cell density of 9 log CFU/mL and anaerobically incubated at 37 °C up to 4 h. The survival rates were determined after 2 and 4 h, as described before. All analyses were performed in triplicate. 

### 2.6. Survival during Simulated Gastrointestinal Transit

The ability of the LAB strains to survive during the gastrointestinal (GI) transit was determined in vitro as described by Pithva et al. [38] with slight modifications. Overnight cultures were centrifuged, and bacterial cells were standardized to 9 log CFU/mL by re-suspending in phosphate buffer solution (PBS). The obtained cell suspension was mixed with the simulated gastric juice (SGJ) and incubated for 2 h at 37 °C, under microaerophilic conditions (agitation 200 rpm). After centrifugation, the bacterial cells were re-suspended in the simulated intestinal fluid (SIF) and incubated at 37 °C for 3 h. SGJ and SGJ–SIF-treated cells were 10-fold diluted and plated on MRS agar for the determination of cell viability. All chemicals were obtained from Sigma Aldrich (St. Louis, MO, USA). Analyses were conducted in triplicate.

### 2.7. Antimicrobial Activity

The LAB strains were tested for antagonistic activity against *E. coli* ATCC 25922, *Staphylococcus aureus* ATCC 6538, *L. monocytogenes* DSM 12464, and *Salmonella enterica* serovar *typhimurium* ATCC 14028 as target bacteria. The assay was performed by the agar spot test following the method previously described [39]. In detail, overnight cultures (7 log CFU/mL) were spotted on MRS agar, and plates were anaerobically incubated at 37 °C for 24 h. After colony growth, plates were overlaid with a microorganism-specific soft agar medium (0.8% *w*/*v*), seeded with 1.0% (*v*/*v*) of an active overnight culture of single target strain (at final concentration of 7 log CFU/mL), and aerobically incubated at 37 °C. After incubation for 48 h, the appearance of inhibition zones around lactobacilli spots was visually detected and, based on diameter size, results were expressed as: (-) no inhibition zone; (+) inhibition zone <10 mm; (++) inhibition zone between 11 and 20 mm; (+++) inhibition zone > 20 mm. Analyses were carried out in triplicate.

### 2.8. Hydrophobicity, Auto-Aggregation, and Co-Aggregation Abilities

The cell surface hydrophobicity (H%) was determined according to Caggia et al. [24]. The auto-aggregation (Auto-A%) and co-aggregation (Co%) abilities were tested according to Solieri et al. [40]. For the co-aggregation assay, *E. coli* 555, *L. monocytogenes* DSM 12464, and *S. enterica* serovar *typhimurium* ATCC 14028 were used as pathogenic strains. All analyses were performed in triplicate. 

### 2.9. Adhesion to Caco-2 Cells

The ability of the LAB strains to adhere to Caco-2 cell lines was evaluated following the method described by Dhanani et al. [41], and results were expressed as adhesion percentage (%). The adhesion assay was repeated three times in three separated experiments.

### 2.10. Antioxidant Activity 

The antioxidant activity was tested by using the 2,2-azino di-(3-ethylbenzthiazoline sulfonate) ABTS assay [42]. A standard curve was obtained using Trolox at different concentrations (20–1000 μM). Results were expressed as μM of Trolox equivalent per liter (μmol TE/L). In addition, the antioxidant activity was also estimated by testing the oxidation of oleic acid, as described by Osawa and Namiki [43] with some modifications [44]. In detail 1 mL of cell-free supernatant was added to 1 mL of 0.1 M PBS (pH 7.0) and 1 mL of linoleic acid (50 mM) in ethanol (99.5%). The oxidation was measured by determining ferric thiocyanate [45]. Butylated hydroxytoluene (BHT) and α-tocopherol (1 mg/mL) were used as positive controls. The negative reference (no antioxidants, un-inoculated MRS broth) was also tested. Analyses were performed in triplicate. 

### 2.11. Anti-Inflammatory Activity 

Human pro-monocytic cells U937 cell line was used as a source of inflammatory response cell [46,47]. U937 cell line was resuspended in a Dulbecco’s modified Eagle’s medium (DMEM) 1 g/L D-glucose (Gibco, Life Technologies, Milan, Italy) supplemented with 10% *v/v* heat-inactivated fetal bovine serum (FBS) (Invitrogen, Carlsbad, CA, USA), 1% penicillin/streptomycin (Carlo Erba, Milan, Italy) antibiotic/antimycotic solution, and 60 mg/mL of gentamicin (Gibco, Monza, Italy). U937 cells were differentiated into macrophages by treatment with 200nM PMA (Phorbol 12-myristate 13-acetate) for 72 h. Cells were divided into 8 groups in relation to the different treatment. In order to simulate the inflammatory process, cells were pre-treated with lipopolysaccharide (LPS) at a concentration of 100 ng/mL for 2 h. The anti-inflammatory effect of LAB strains was evaluated treating the groups with LAB strains’ broth culture at the concentration of 10 μg/mL for 6 h. At the end of the treatment, cells were washed with PBS, collected by trypsinization, and then lysed for RNA extraction. All analyses were performed in triplicate. 

### 2.12. RNA Extraction and qRT-PCR

RNA was extracted by Trizol reagent kit (Invitrogen, Carlsbad, CA, USA) following the manufacturer instructions. Total RNA was extracted from 2.8 × 10^6^ U937 cells. One μg of total RNA was used to first strand cDNA, which was then synthesized with Applied Biosystem reverse transcription reagent (Foster City, CA, USA). 

Quantitative real-time PCR (qRT-PCR) was performed in 7900HT Fast Real-Time PCR System Applied Biosystems using the SYBR Green PCR MasterMix (Life Technologies), using the primer sequences showed in Table S1. qRT-PCR was performed using SYBR Green PCR MasterMix (Life Technology, Milan, IT) 5 μL, Forward Primer 0.2 μL, Reverse Primer 0.2 μL, UltraPureTM Distilled Water DNase/RNase Free (Invitrogen by Life Technologies, Milan, Italy) 4.1 μL, and cDNA 1 μL. The amplified genes were: COX-1 (Ciclooxygenase-1), COX-2, GAPDH (Glyceraldeyde-3-phosphate dehydrogenase), IL-8 (Interleukin-8), and IL-10. The specific PCR products were detected by the fluorescence of SYBR Green, the double-stranded DNA binding dye. PCR reactions were subjected to 40 cycles of 90 °C for 20 s, 95 °C for 3 s and 60 °C for 30 s. The relative mRNA expression level was calculated by the threshold cycle (Ct) value of each PCR product and normalized with that of GAPDH by using comparative 2^-ΔΔCt^ method. The analysis was performed in triplicate.

### 2.13. Preliminary Identification of Metabolites Responsible for Antagonistic Activity against Pathogens 

To elucidate the nature of substances responsible for the antagonistic activity against pathogens, cell-free supernatants (CFSs) of the *Lactobacillus* strains selected as reported above were obtained by centrifugation in overnight cultures (7000× *g*, 15 min, 5 °C) and further sterilization by filtration through a 0.22-µm pore filter (Millipore, Italy). CFSs were differently treated according to dos Santos and co-workers [48]. The antagonistic activity was evaluated by measuring the diameter of inhibition zones and was determined in triplicate. 

### 2.14. Detection of Genes for Virulence and Antibiotic Resistance

The lactobacilli strains, screened as reported above, were subjected to a PCR-based approach in order to investigate the presence of genes encoding for gelatinase (*gel*E), hyaluronidase (*hyl*), aggregation substance (*asa*1), enterococcal surface protein (*esp*), cytolisin (*cyl*A), endocarditis antigen (*efa*A), and adhesion of collagen (*ace*) using the primer pairs and conditions previously described [48]. *Enterococcus faecalis* ATCC 29212 was used as positive control. In addition, the presence of genes related to resistance to erythromycin (*erm*A, *erm*B, and *erm*C), tetracycline (*tet*K, *tet*L, *tet*M, *tet*O, and *tet*S), gentamycin (*aac*(6′)-Ie-*aph*(2″)-Ia), chloraphenicol (*cat*A), and aminoglycosides-type antibiotics (*aph*(3′)-IIIa, *ant*(4′)-Ia, *aph*(2″)-Id, *aph*(2″)-Ic, *aph*(2″)-Ib, *ant*(6)-Ia) was evaluated. The primers pairs and PCR conditions used were according to Todorov and co-workers [49]. Generated amplicons were separated on 0.8 to 2.0% (*w*/*v*) agarose gels in 0.5 × TAE buffer using GelRed^®^ Nucleic Acid Gel Stain (Biotium, Fremont, CA, USA) [49].

### 2.15. Statistical Analysis

All data were expressed as a mean ± SD of triplicate experiments. Data related to hydrophobicity, auto-aggregation, co-aggregation, adhesion to Caco-2 cells abilities, antioxidant, and RT-PCR data were normalized and subjected to one-way ANOVA followed by Tukey’s multiple comparison test using GraphPad Prism 6 software, and differences were considered statistically significant when *P* < 0.05. 

## 3. Results

### 3.1. LAB Isolation and Identification

**Figure 1:**
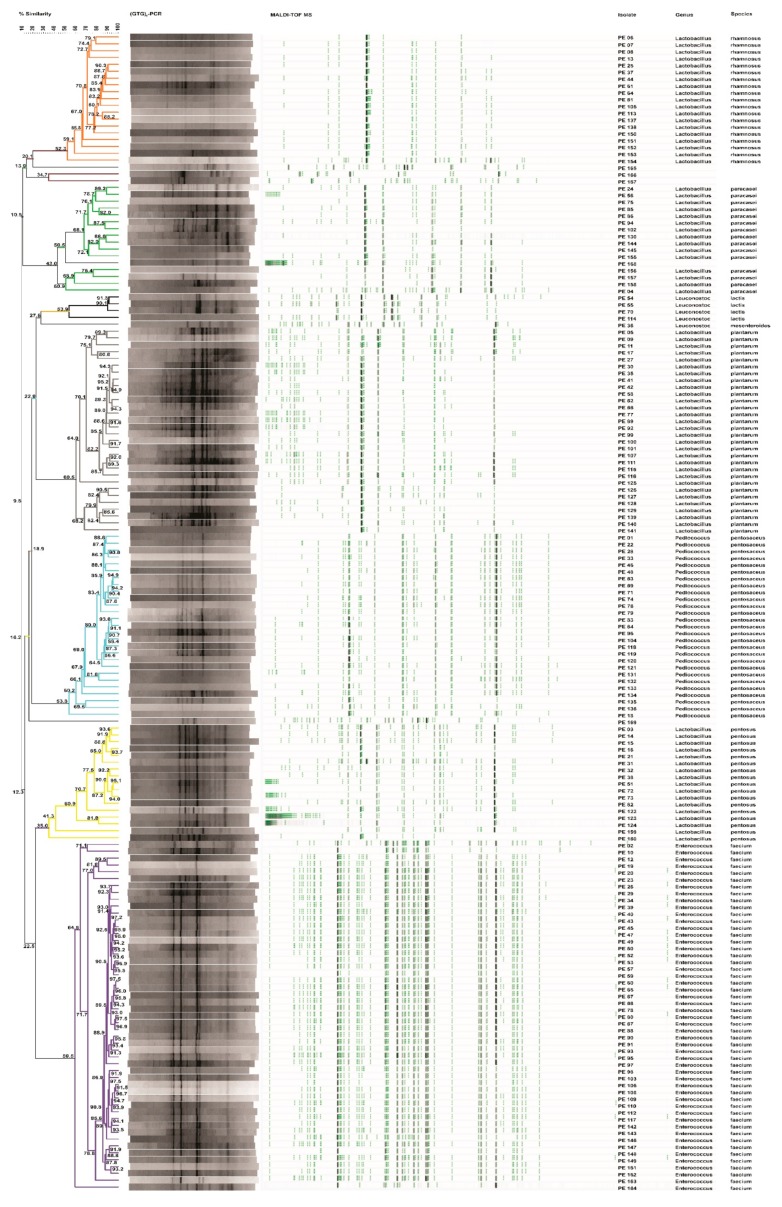
UPGMA dendrogram of the polyphasic approach combining Rep-PCR and MALDI-TOF MS analyses of presumptive lactic acid bacteria (LAB) isolated from Piacentinu Ennese (PE) cheese samples. Node values indicate the average percentage of similarity based on (GTG)_5_-PCR and MALDI-TOF MS profiles. The tree was made with BioNumerics version 5.1.

One hundred and sixty-nine isolates from MRS agar plates were considered LAB based on their positive Gram reaction, non-motility, absence of catalase activity, and spore formation and rod or coccal shape. Genotypic identification through Rep-PCR coupled to MALDI-TOF MS analysis allowed to identify eight species (Figure 1). The isolates were identified as *Enterococcus faecium* (30%), *Lactobacillus plantarum* (18%), *Pediococcus pentosaceus* (16%), *Lactobacillus rhamnosus* (11%), *Lactobacillus pentosus* (10%), *Lactobacillus paracasei* (9%), *Leuconostoc lactis* (2%), and *Leuconostoc mesenteroides* (1%). Five isolates (3%), not identified by MALDI-TOF MS analysis, were ascribed to *Enterococcus durans* species by 16S rRNA gene sequencing, with an identity percentage (%) of 98%. One hundred and thirteen (113) LAB strains, except those ascribed to *E. faecium* and *E. durans* species, not included in the Qualified Presumption of Safety (QPS) list, were investigated for probiotic traits.

### 3.2. Safety Properties

None of the tested LAB strains showed the ability to produce DNase and gelatinase, to exert hemolytic activity, or to degrade the mucin. Beyond the aforementioned safety properties, the ability of strains to produce biogenic amines (BA) was tested. Results showed that none of the 113 tested strains were able to produce BA, except the *Luec. mesenteroides* PE36 strain, which produced tyramine (data not shown). 

The 112 strains fulfilling the above safety requirements were studied for antibiotic susceptibility. Overall, 94% of them (105/112) were sensitive to all the tested antimicrobials. Based on the EFSA breakpoints, no antibiotic resistance was found among strains belonging to *L. plantarum*, *L. pentosus*, *L. rhamnosus*, and *L. paracasei* species. Resistance to kanamycin was exhibited by *Leuc. lactis* PE54 and PE114 strains, while *P. pentosaceus* PE18, PE28, PE83, PE76, and PE22 strains were found to be streptomycin-resistant (Table S2). 

### 3.3. Lysozyme Tolerance

**Figure 2:**
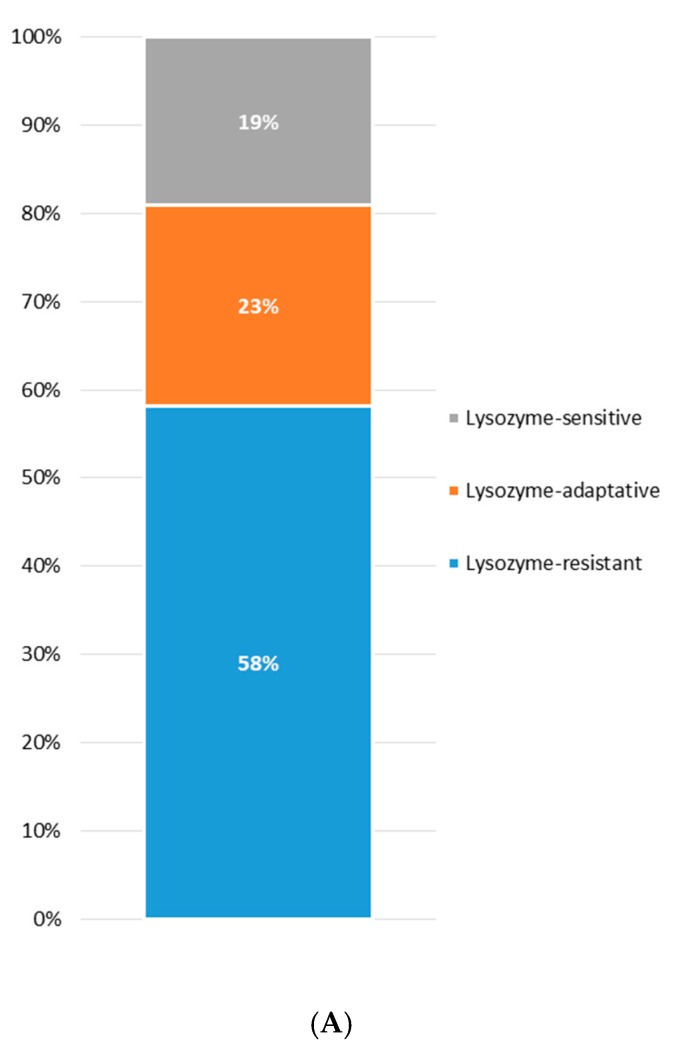

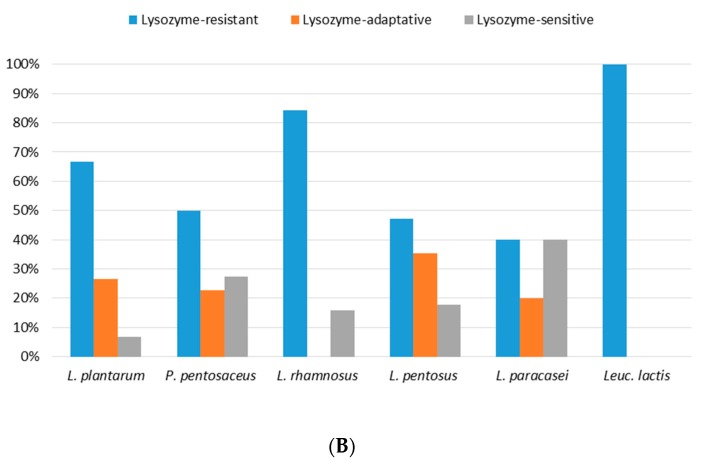
Occurrence of lysozyme tolerance among the screened strains (**A**) and its distribution within each species (**B**).

The occurrence of lysozyme tolerance among the screened strains and its distribution within each species are shown in Figure 2A,B, respectively. Overall, the majority of the strains were categorized as lysozyme-resistant (58%) showing a survival rate higher than 90% after both 30 and 120 min. The 23% was considered lysozyme-adaptive, since after an initial decrease in viable cells (about 1 log unit), a slight increase was observed after 120 min of exposure to lysozyme. Only 19% of the strains showed a significant reduction in viability (more than 3 log units) after both 30 and 120 min of exposition to lysozyme and were classified as lysozyme-sensitive (Figure 2A). Zooming on lysozyme tolerance within each species, *Leuc. lactis*, *L. rhamnosus*, and *L. plantarum* harbored the highest percentages of lysozyme-resistant strains, while *L. paracasei* and *P. pentosaceus* gathered the highest proportions of sensitive strains (Figure 2B).

### 3.4. Acidic Tolerance

**Figure 3:**
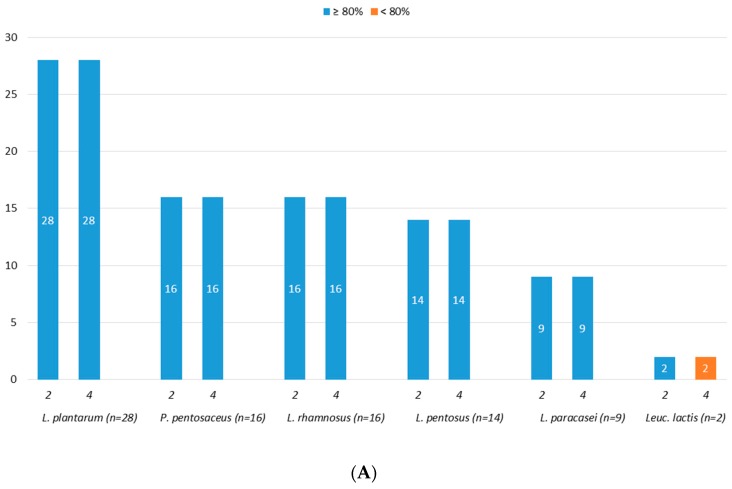

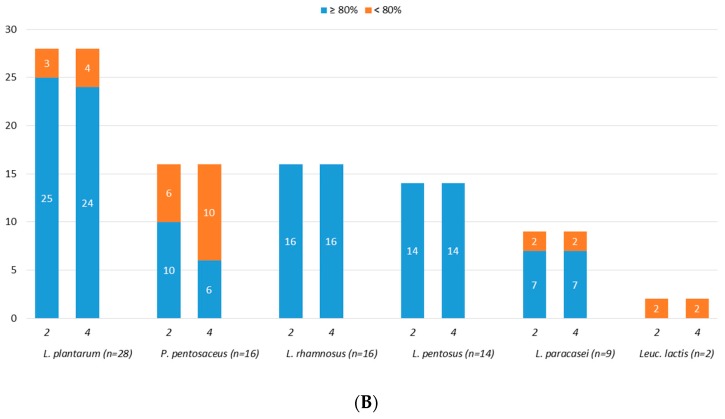
Number of strains showing survival rates ≥80% or <80% after 2 and 4 h of incubation at pH 3.0 (**A**) and at pH 2.0 (**B**).

Eighty-five (85) strains were screened for acidic tolerance and results, reported as number of tolerant strains, as shown in Figure 3A,B. Overall, starting from an initial number of viable cells (control cells) of about 9.0 log CFU/mL, good acidic tolerance was observed at both pH 3.0 and pH 2.0. In detail, all the tested strains, with the exception of two *Leuc. lactis* (PE55 and PE70), showed survival rate higher than 80% after exposure to pH 3.0 for 4 h (Figure 3A). As reported in Figure 3B, the exposure to pH 2.0 for both 2 and 4 h had no effect on strains belonging to *L. rhamnosus* and *L. pentosus* species, while it affected the survival rate of four *L. plantarum* (PE05, PE35, PE62, PE111), ten *P. pentosaceus* (PE01, PE33, PE46, PE48, PE63, PE69, PE71, PE74, PE79, PE84), and two *L. paracasei* (PE56, PE94) strains, which showed a survival rate lower than 80%. 

### 3.5. Bile Salts Tolerance 

**Figure 4:**
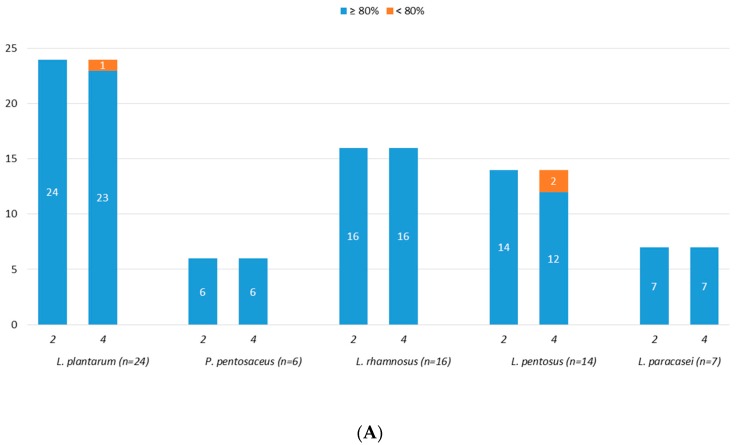

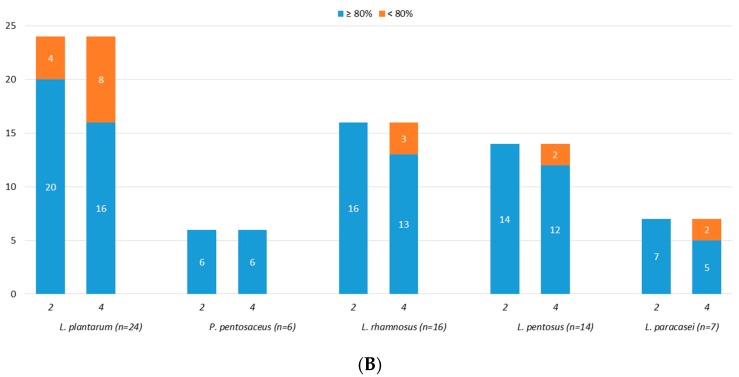
Number of strains showing survival rates ≥80% or < 80% after treatment, for 2 and 4 h, with 0.5% (**A**) and 1.0% (**B**) of bile salts.

Results of tolerance to bile salts are shown in Figure 4A,B. Overall, among the 67 tested strains, 0.5% (*w*/*v*) of bile salts affected only one *L. plantarum* strain (PE17) and two *L. pentosus* strains (PE03 and PE51), whereas at 1.0% (*w*/*v*) of concentration the 94% (63/67) and the 78% (52/67) of the strains displayed bile tolerance after 2 and 4 h, respectively.

### 3.6. Survival During In Vitro GI Transit 

The selected strains, screened as above, were evaluated for their survivability during passage through the GIT, and results are reported in Table 1. Overall, 61% (32/52) of the strains showed good tolerance to both simulated gastric juice (SGJ) and simulated intestinal fluid (SIF). Twenty strains exhibited a strong reduction in viability (more than 3 log units) after the exposure to SGJ. *L. paracasei* strains were not affected by simulated gastric digestion (Table 1).

### 3.7. Antagonistic Activity against Pathogens 

The antagonistic activity against food spoilage and pathogenic bacteria displayed by the 32 selected strains is reported in Table 2. The applied in vitro method allowed a rapid screening of the studied population. Overall, *L. rhamnosus*, *L. pentosus* and *L. paracasei* strains showed antagonistic activity against the tested pathogens, while none of the *P. pentosaceus* strains inhibited the growth of the pathogens. *L. rhamnosus* PE44, PE61, PE25, and PE13 strains, as well as *L. paracasei* PE24, PE85, and PE86 strains inhibited all tested pathogens, generating inhibition zones larger than 10 mm (Table 2, Figure S1). 

### 3.8. Hydrophobicity, Auto-Aggregation, and Co-Aggregation Abilities 

Table 3 summarizes the surface characteristics (hydrophobicity, auto-aggregation, and co-aggregation) of the 32 selected lactobacilli strains. Overall, the vast majority of the strains showed cell surface hydrophobicity higher than 50%, except *L. plantarum* strains, which displayed values ranging from 23% to 41%. In detail, *L. rhamnosus* PE13, PE25, PE44, and PE61 strains, together with *L. paracasei* PE24, PE85, and PE86 strains showed hydrophobicity higher than 70% (Table 3). All tested lactobacilli strains, with the exception of *L. plantarum* strain PE27, *L. pentosus* strains PE21, PE72, and PE73 showed good auto-aggregation ability, with values ranging from 53% to 77% (Table 3). The highest Auto-A% was recorded by both *L. rhamnosus* and *L. paracasei* strains. Regarding co-aggregation with pathogens, a broad range of variation was detected, with *L. rhamnosus* and *L. paracasei* strains showing co-aggregation values higher than 50% (Table 3).

### 3.9. Adhesion to Caco-2 Cells 

**Figure 5:**
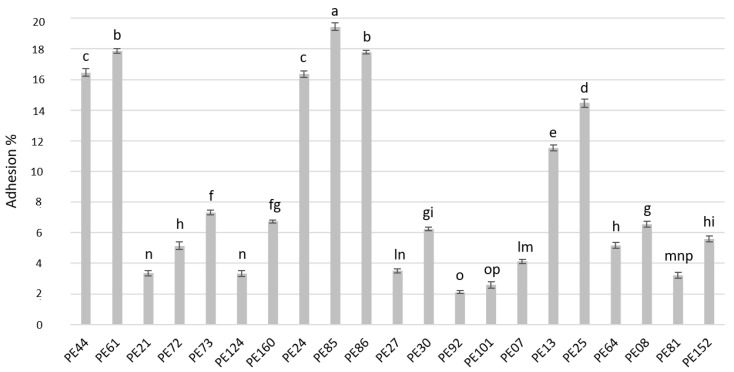
Adhesion (%) of lactobacilli to Caco-2 cells. Data are the means from three independent experiments. Bars represent standard deviations. Letters a–p indicate *P* < 0.05.

The adhesion ability of the selected *Lactobacillus* strains to Caco-2 cells is shown in Figure 5. Overall, the adhesion capacity was strain-dependent. *L. rhamnosus* strains PE44, PE61, PE25, and PE13 and *L. paracasei* strains PE24, PE85, and PE86 exhibited the highest binding ability.

### 3.10. In Vitro Antioxidant Activity 

**Figure 6:**
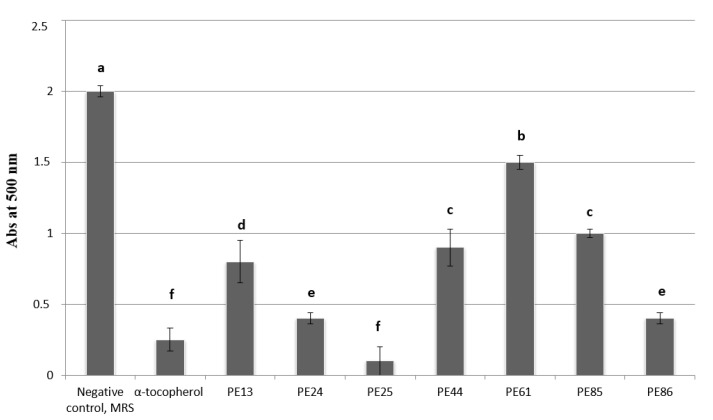
Lipid peroxidation inhibitory activity (Abs at 500 nm) in cell free supernatants (CFSs) of lactobacilli grown in MRS broth or un-inoculated and incubated MRS broth (control) for 24 h at 30 °C. The activity was measured under a linoleic acid oxidation system for 8 days. α-tocopherol (1 mg/mL) was used as the positive controls. Data are the means from three independent experiments. Bars represent standard deviations. Letters a–f indicate *P* < 0.05.

The total antioxidant capacity of the seven selected lactobacilli strains (PE13, PE24, PE25, PE44, PE61, PE85, PE86) was determined based on the scavenging activity towards radical cation 2,2′-azino-di-[3-ethylbenzthiazoline sulfonate] (ABTS). All lactobacilli showed the ability to inhibit the peroxidation of linoleic acid (Figure 6). Among them, the PE25 strain showed the highest antioxidant activity which was similar (*P* < 0.05) to those found for the well-known antioxidant compound (α-tocopherol).

### 3.11. In Vitro Anti-Inflammatory Activity

In order to investigate the anti-inflammatory effect of the selected lactobacilli strains, the gene expression of COX-1, COX-2, IL-8, and IL-10 was evaluated. To in vitro simulate the inflammation conditions, the treatment with strains’ broth culture was carried out on differentiated macrophages stellate cells treated with LPS. Table 4 shows the gene levels expression in differentiated human macrophages after treatment with LPS. In detail, the expression of genes was significantly modified compared to untreated cells. In particular, in the U937 cell line, three strains (PE25, PE44, PE61) downregulated both COX-1 and COX-2, while all the other stains, with the exception of PE85, were able to downregulate only COX-2. Regarding the expression levels of IL-8 and IL-10, results showed that all strains upregulated IL-10, whereas seven strains (PE24, PE25, PE44, PE61, PE85, and PE86) reduced the expression levels of the IL-8 gene.

### 3.12. Preliminary Identification of Metabolites Responsible for Antagonistic Activity against Pathogens 

To determine the type of the inhibitory substances (e.g., bacteriocins, organic acids, and/or hydrogen peroxide) responsible for the antimicrobial activity against pathogens, specific assays were performed. The treatment of CFSs with proteases did not result in any changes of antimicrobial activity, indicating that the antagonism was not due to bacteriocins production or protein-based compounds. The same results were achieved after treating the CFSs with catalase, suggesting that hydrogen peroxides production was not responsible for pathogens inhibition. Differently, after CFSs neutralization none of the tested strains showed antagonistic activity against pathogens, suggesting that the produced organic acids were involved in the observed antagonistic activity.

### 3.13. Virulence Factors and Antibiotic Resistance Genes

The seven strains (PE24, PE25, PE44, PE61, PE85 and PE86) did not show the presence of genes encoding for gelatinase, hyaluronidase, aggregation substance, enterococcal surface protein, cytolisin, endocarditis antigen, and adhesion of collagen (Table S3). In addition, the PCR-based approach did not reveal the presence of genes related to resistance to antibiotics (erythromycin, tetracycline, gentamycin, chloraphenicol, and aminoglycosides-type antibiotics) (Table S3). 

## 4. Discussion

Spontaneously fermented milk products are produced in many parts of the world and constitute an excellent source of microorganisms with health-promoting properties, particularly bacteria from the genus *Lactobacillus*. Scientists and ISAPP (International Scientific Association for Probiotics and Prebiotics) experts have emphasized the importance of the connection between eating fermented foods and human health. In particular, many regional cheeses in Europe have already been used to isolate promising probiotic bacteria [24,40,48,50] demonstrating that fermented dairy foods could have a potential positive impact on human health [51,52,53,54]. Piacentinu Ennese PDO (PE) is an artisanal cheese produced in Southern Italy, without the addition of starter cultures and characterized by a complex microbiota which arises from milk, pasture in animal diet, environment, and equipment used during the cheese making [55]. According to previous reports describing the LAB profile of different raw milk cheeses produced under traditional practices, in PE cheese lactobacilli dominated the non-starter lactic acid bacteria (NSLAB) population [24,40,50,56,57,58,59]. In addition, *Enterococcus faecium* [25,30,32] along with *Pediococcus pentosaceus*, *Leuconostoc mesenteroides* and *Leu. lactis* species were also detected as part of the dominant cheese microbiota [60,61,62]. 

In the present study, 113 LAB strains, isolated from PE cheese, were in vitro screened for safety and functional properties, as recommended by the FAO/WHO [10]. Although in vitro data alone are insufficient to define a strain as probiotic, they are still valuable and can provide scientific insight into specific characteristics of the potential probiotic strain. Thus, a series of in vitro tests can refine the selection of suitable stains [63]. 

Generally, resistance to gastro-intestinal digestion, antimicrobial activities, auto-aggregation, co-aggregation, hydrophobicity, adhesion to epithelial cells, and antibiotic susceptibility are the main features qualifying a microorganism as probiotic [64]. Among the aforementioned properties, antibiotic resistance is a topic of concern due to the risk of carry resistance from food to the GIT bacterial population [65]. Although LAB have a long history of safe use, antibiotic resistance to some antimicrobials is still controversial and should, therefore, be addressed for each strain. More recently, increasing attention has been given to antimicrobial use in farm animal feeding, which can act as a precursor to antibiotic-resistant microorganisms in humans [40]. It was well established that chloramphenicol and tetracycline are emerging transferable resistant genes among lactobacilli [66,67,68,69,70,71,72,73]. Our data, based on phenotypic approach, revealed that the majority of the strains were sensitive to the antibiotics reported above, suggesting that probably PE cheese is produced in a geographical area where no systematic use of antibiotics as growth promoters is carried out, in accordance with previous works [40,74]. Regarding kanamycin and streptomycin, in accordance with previous reports [75,76,77,78], *Leuc. lactis* and *P. pentosaceus* strains were resistant, suggesting that the cut-off of streptomycin and kanamycin for *Leuconostoc* and *Pediococcus* should be updated. 

Along with susceptibility to antimicrobials, the absence of DNase, gelatinase, hemolytic activity, mucin degradation, and BA production abilities attested the safety of the studied strains, with the exception of only one *Leuc. mesenteroides* strain which was able to produce tyramine. The ability of *Leuconostoc* strains isolated from cheese to decarboxylate tyrosine, histidine, ornithine, and lysine was previously reported [79]. In addition, the absence of genes encoding for virulence factors and antibiotic resistance confirmed the safety properties of the seven selected strains, excluding the risk of delivering virulence factors to the host. 

Beyond the safety requirements, probiotic candidates were in vitro subjected to the harsh environmental conditions of the GI tract, (e.g., salivary lysozyme, gastric acidity, and bile salts), which can drastically reduce the bacterial survivability during the GI passage [64]. According to previous data [15,24,40,80], a high in vitro tolerance to lysozyme and to bile salts was detected, even a strain specific survival pattern was achieved among isolates belonging to the same species. 

In order to deliver a health benefit to the host, probiotic candidates should be able to adhere and colonize the GIT tract interacting with the resident bacteria. Several mechanisms are involved in the bacterial adherence, which depend on cell surface properties and extracellular bacterial protein profile. It is generally agreed that auto-aggregation may increase the colonization potential of lactobacilli while hydrophobicity, related to the presence of hydrophobic molecules in the cell surface [81,82,83], may strengthen the capacity to adhere to the intestinal mucosa supporting the contact between microorganism and host cell. Even if some studies suggested that probiotic adhesion might be cell surface hydrophobicity-dependent, there is still no consensus since no correlation between hydrophobicity and bacterial adhesion has been clearly observed [49]. In the present work, the selected lactobacilli exhibited hydrophobicity properties along with adhesion ability to Caco-2 cells.

One of the most notable function of probiotic strains is to antagonize harmful microorganisms preventing foodborne infections and the growth of enteric pathogens through different mechanisms, such as the production of antimicrobial compounds, the competition for nutrients and growth factors, as well as by binding the receptor sites [84]. In addition, the ability to co-aggregate with pathogens is considered a positive trait, supporting the formation of a physical-chemical barrier that prevents pathogen colonization. *L. rhamnosus*, and *L. paracasei* strains, here investigated, showed a broad spectrum of activity against the tested foodborne and intestinal pathogens along with high percentage of co-aggregation. In addition, as previously reported [24,38,85], a strain-specific antimicrobial activity was confirmed, and the inhibitory effect showed by the selected seven strains was due to acidic compounds of supernatant. 

It is interesting to underline also the strain-specific property of the selected strains related to antioxidant activity, revealing the good potential of the PE25 strain to inhibit the linoleic acid peroxidation. Antioxidant activity has been largely described for lactobacilli [44,86,87], and oxidative stress and lipid peroxidation are believed to play a significant role in the development of tissue damage and in several human pathologies [88]. 

In order to evaluate the anti-inflammatory effect, the gene expression of inflammatory biomarkers, in an in vitro model consisting of human macrophages exposed to LPS, was also evaluated. The combined treatments of strains’ broth culture with LPS showed the inhibition of inflammation. In particular, COX-1 and -2 genes were upregulated by LPS treatment while tested strains were able to counteract this effect. COX1 and COX2 are molecules that catalyze prostaglandin biosynthesis and are targets of non-steroidal anti-inflammatory drugs [89,90,91]. However, the obtained results showed that all tested strains, with the exception of one (PE13), were able to reduce the inflammatory cytokine IL8 and to increase the anti-inflammatory cytokine IL10. These results indicate that the analyzed stains, as well as having inhibitory activity of cyclooxygenase, are skilled also in regulating the expression of cytokines helping to reduce the recruitment of immune organism cells. 

The aforementioned preliminary data allowed us to validate the potential probiotic properties of the selected lactobacilli strains suggesting the Piacentinu Ennese PDO cheese as reservoir of promising probiotic bacteria. Further studies will be done in order to characterize the organic acids (such as lactic, butyric, acetic, citric, succinic, glutamic acids) responsible for the antagonistic activity against pathogens. In addition, the in vitro data will be in vivo validated in a cohort of patients with functional gastrointestinal diseases. 

## 5. Conclusions

The present study revealed that Piacentinu Ennese PDO represents a good source of *L. rhamnosus* and *L. paracasei* strains with promising in vitro probiotic features. Based on these results, further studies will be done to characterize the promising strains for technological properties in order to be used in functional food. In addition, whole genome sequencing-based approaches could be applied in order to explore the potential beneficial effects of the selected strains on human health.

## Figures and Tables

**Table 1:** Survival (log CFU/mL) of the tested strains during in vitro gastrointestinal transit.

Species	Strain	CI	SGJ	SIF
*L. plantarum* (*n* = 16)	PE09	9.03 ± 0.11	5.30 ± 0.02	5.11 ± 0.02
	PE11	9.12 ± 0.03	8.96 ± 0.06	8.84 ± 0.02
	PE27	9.10 ± 0.07	8.02 ± 0.03	8.06 ± 0.06
	PE30	9.01 ± 0.14	8.11 ± 0.02	8.01 ± 0.02
	PE41	9.16 ± 0.02	4.98 ± 0.05	4.16 ± 0.03
	PE42	9.28 ± 0.02	5.26 ± 0.08	5.15 ± 0.06
	PE58	9.21 ± 0.05	5.02 ± 0.02	4.00 ± 0.03
	PE66	9.22 ± 0.03	8.14 ± 0.06	8.03 ± 0.05
	PE77	9.31 ± 0.06	5.31 ± 0.02	5.03 ± 0.08
	PE92	9.24 ± 0.08	8.16 ± 0.02	8.11 ± 0.03
	PE99	9.18 ± 0.05	9.01 ± 0.03	8.86 ± 0.06
	PE101	9.20 ± 0.11	8.28 ± 0.04	8.14 ± 0.02
	PE107	9.30 ± 0.13	4.05 ± 0.02	3.83 ± 0.02
	PE115	9.12 ± 0.02	6.00 ± 0.06	5.84 ± 0.03
	PE139	9.06 ± 0.12	4.21 ± 0.08	4.07 ± 0.10
	PE141	9.19 ± 0.05	5.11 ± 0.02	5.03 ± 0.02
*L. rhamnosus* (*n* = 13)	PE07	9.13 ± 0.04	8.12 ± 0.07	8.06 ± 0.08
	PE08	9.11 ± 0.03	8.13 ± 0.04	8.02 ± 0.03
	PE13	9.06 ± 0.07	8.01 ± 0.04	8.00 ± 0.02
	PE25	9.14 ± 0.05	8.20 ± 0.06	8.15 ± 0.02
	PE44	9.19 ± 0.07	8.01 ± 0.08	7.93 ± 0.06
	PE61	9.10 ± 0.04	8.00 ± 0.05	7.96 ± 0.04
	PE64	9.26 ± 0.06	8.87 ± 0.02	8.51 ± 0.04
	PE81	9.23 ± 0.08	8.68 ± 0.02	8.05 ± 0.06
	PE113	9.36 ± 0.04	4.88 ± 0.06	3.12 ± 0.04
	PE137	9.07 ± 0.03	5.03 ± 0.06	4.91 ± 0.03
	PE138	9.03 ± 0.11	5.21 ± 0.12	5.10 ± 0.07
	PE151	9.23 ± 0.05	5.23 ± 0.11	5.06 ± 0.10
	PE152	9.22 ± 0.07	9.04 ± 0.08	8.93 ± 0.06
*L. pentosus* (*n* = 12)	PE14	9.15 ± 0.04	8.11 ± 0.03	8.04 ± 0.03
	PE15	9.11 ± 0.03	8.02 ± 0.04	7.96 ± 0.06
	PE16	9.23 ± 0.11	4.98 ± 0.07	4.33 ± 0.07
	PE21	9.17 ± 0.06	9.03 ± 0.04	8.96 ± 0.07
	PE38	9.11 ± 0.03	5.12 ± 0.08	4.91 ± 0.07
	PE72	9.07 ± 0.08	8.03 ± 0.07	7.94 ± 0.08
	PE73	9.09 ± 0.07	8.21 ± 0.06	8.07 ± 0.07
	PE122	9.11 ± 0.06	8.18 ± 0.07	8.09 ± 0.04
	PE123	9.16 ± 0.03	5.54 ± 0.08	5.31 ± 0.03
	PE124	9.09 ± 0.06	8.86 ± 0.12	8.03 ± 0.06
	PE159	9.21 ± 0.11	5.18 ± 0.14	5.03 ± 0.04
	PE160	9.18 ± 0.04	8.07 ± 0.04	7.91 ± 0.07
*P. pentosaceus* (*n* = 6)	PE96	9.16 ± 0.08	8.25 ± 0.02	8.13 ± 0.02
	PE104	9.07 ± 0.02	8.15 ± 0.04	7.95 ± 0.03
	PE120	9.30 ± 0.10	8.21 ± 0.03	8.02 ± 0.02
	PE133	9.28 ± 0.02	5.24 ± 0.02	5.21 ± 0.04
	PE135	9.18 ± 0.04	5.02 ± 0.02	4.56 ± 0.06
	PE136	9.15 ± 0.06	4.34 ± 0.02	4.46 ± 0.02
*L. paracasei* (*n* = 5)	PE04	9.16 ± 0.06	9.07 ± 0.06	8.94 ± 0.06
	PE24	9.09 ± 0.07	8.24 ± 0.07	8.10 ± 0.03
	PE85	9.13 ± 0.11	8.16 ± 0.14	7.98 ± 0.07
	PE86	9.20 ± 0.07	8.83 ± 0.07	8.03 ± 0.14
	PE102	9.14 ± 0.03	8.96 ± 0.07	8.84 ± 0.04

Cell density before transit (CI) and upon sequential exposure to simulated gastric juice (SGJ) and simulated intestinal fluid (SIF), determined by viable counting on Man, Rogosa and Sharpe (MRS) agar. Data are shown as mean log CFU/mL of three independent experiments and standard deviation.

**Table 2:** Antimicrobial activity against food spoilage and pathogenic bacteria.

Species	Strains	*E. coli* ATCC 25922	*S. aureus* ATCC 6538	*L. monocytogenes* DSM 12464	*S. enterica s. typhimurium* ATCC 14028
*L. rhamnosus* (*n* = 9)	PE44, PE61, PE25, PE13	+++	++	+++	+++
	PE64, PE08, PE81	++	-	+++	++
	PE07	-	++	-	+
	PE152	++	++	-	-
*L. pentosus* (*n* = 8)	PE14, PE15, PE21	+++	++	++	+++
	PE72, PE73	+++	+++	-	+
	PE124, PE160, PE122	-	-	-	-
*L. plantarum* (*n* = 7)	PE30	+	-	-	-
	PE92, PE101, PE11	-	-	-	-
	PE66	-	+	-	+
	PE09, PE27	+	+	-	-
*L. paracasei* (*n* = 5)	PE24, PE85, PE86	++	+++	++	++
	PE04, PE102	-	-	-	
*P. pentosaceus* (*n* = 3)	PE96, PE104	-	-	-	-
	PE120	-	-	-	-

(-) no inhibition zone; (+) inhibition zone <10 mm; (++) inhibition zone 11–20 mm; (+++) inhibition zone > 20 mm.

**Table 3:** Surface properties of the subset of 32 lactobacilli strains.

Species	Strains	H%	Auto-A%	CoA%		
				*E. coli* 555	*L. monocytogenes*	*S. enterica* *S. typhimurium*
*L. plantarum*	PE27	28.13 ± 0.54 ^a^	12.30 ± 0.19 ^a^	14.20 ± 0.20 ^c^	23.22 ± 0.21 ^e^	24.31 ± 0.26 ^e^
	PE30	31.09 ± 0.19 ^b^	53.13 ± 0.15 ^e^	21.47 ± 0.27 ^d^	32.54 ± 0.20 ^h^	12.25 ± 0.15 ^b^
	PE92	41.21 ± 0.38 ^d^	56.30 ± 0.23 ^g^	46.18 ± 0.12 ^h^	14.38 ± 0.22 ^b^	37.17 ± 0.10 ^h^
	PE101	36.30 ± 0.16 ^c^	58.41 ± 0.38 ^h^	31.48 ± 0.27 ^f^	23.20 ± 0.18 ^e^	28.63 ± 0.18 ^g^
*L. rhamnosus*	PE07	43.12 ± 0.30 ^e^	74.11 ± 0.14 ^pq^	59.23 ± 0.07 ^no^	58.29 ± 0.24 ^p^	67.33 ± 0.18 ^r^
	PE13	71.36 ± 0.42 ^m^	77.41 ± 0.23 ^s^	51.29 ± 0.16 ^i^	60.33 ± 0.18 ^rr^	52.28 ± 0.13 ^i^
	PE25	78.14 ± 0.13 ^q^	71.37 ± 0.28 ^n^	68.27 ± 0.16 ^r^	72.35 ± 0.19 ^v^	58.26 ± 0.15 ^m^
	PE64	56.39 ± 0.33 ^l^	65.20 ± 0.20 ^l^	54.26 ± 0.13 ^l^	67.34 ± 0.27 ^s^	52.42 ± 0.14 ^i^
	PE08	46.24 ± 0.31 ^f^	69.03 ± 0.23 ^m^	51.17 ± 0.12 ^i^	52.37 ± 0.15 ^m^	60.22 ± 0.19 ^o^
	PE81	41.14 ± 0.37 ^d^	59.99 ± 0.27 ^i^	58.59 ± 0.20 ^n^	53.34 ± 0.24 ^n^	60.30 ± 0.20 ^o^
	PE152	48.35 ± 0.35 ^g^	72.29 ± 0.09 ^o^	63.24 ± 0.18 ^p^	59.37 ± 0.28 ^q^	56.37 ± 0.22 ^l^
	PE44	72.23 ± 0.20 ^mn^	71.47 ± 0.35 ^no^	72.35 ± 0.23 ^t^	51.41 ± 0.17 ^l^	58.64 ± 0.15 ^mn^
	PE61	74.29 ± 0.26 ^no^	76.04 ± 0.34 ^r^	57.27 ± 0.16 ^m^	42.93 ± 0.32 ^i^	59.27 ± 0.23 ^n^
*L. pentosus*	PE21	52.11 ± 0.10 ^i^	19.24 ± 0.17 ^c^	32.27 ± 0.16 ^g^	28.18 ± 0.12 ^f^	24.16 ± 0.12 ^e^
	PE72	56.33 ± 0.26 ^l^	15.16 ± 0.19 ^b^	14.20 ± 0.12 ^c^	29.34 ± 0.21 ^g^	21.73 ± 0.23 ^d^
	PE73	48.22 ± 0.15 ^g^	23.38 ± 0.24 ^d^	6.35 ± 0.20 ^a^	12.18 ± 0.12 ^a^	18.71 ± 0.12 ^c^
	PE124	46.35 ± 0.45 ^f^	54.61 ± 0.28 ^f^	23.40 ± 0.28 ^e^	18.21 ± 0.18 ^d^	26.74 ±0.22 ^f^
	PE160	50.01 ± 0.53 ^h^	57.17 ± 0.13 ^g^	11.26 ± 0.16 ^b^	15.29 ± 0.24 ^c^	9.37 ± 0.20 ^a^
*L. paracasei*	PE24	76.37 ± 0.35 ^p^	76.37 ± 0.24 ^r^	71.48 ± 0.21 ^s^	71.29 ± 0.18 ^u^	61.33 ± 0.11 ^p^
	PE85	73.25 ± 0.24 ^no^	73.23 ± 0.19 ^p^	66.34 ± 0.22 ^q^	69.27 ± 0.17 ^t^	71.19 ± 0.13 ^s^
	PE86	74.33 ± 0.34 ^no^	74.36 ± 0.22 ^q^	59.60 ± 0.20 ^o^	56.39 ± 0.26 ^o^	63.28 ± 0.13 ^q^

Results are expressed as average value and standard deviation of three separate experiments. Different letters in the same column indicate significant differences by one-way ANOVA test, followed by Tukey post-hoc test (*P* < 0.05). H%: Hydrophobicity; Auto-A%: Auto-aggregation; CoA%: Co-aggregation.

**Table 4:** Gene levels expression in differentiated human macrophages.

	COX-1	COX-2	IL-8	IL-10
LPS	2.07 ± 0.10	15.22 ± 0.76	0.64 ± 0.03	128.50 ± 6.42
PE13	34.31 ± 1.71 *	3.24 ± 17.81 *	1.42 ± 0.07 *	103192 ± 5159 *
PE24	24.05 ± 1.20 *	0.09 ± 0.004 *	0.60 ± 0.03	615.96 ± 30.79 *
PE25	1.18 ± 0.21	2.28 ± 0.06 *	0.003 ± 0.02 *	9153 ± 12.1 *
PE44	0.42 ± 0.02 *	2.41 ± 0.12 *	0.0018 ± 9.27 × 10^−5^ *	488 ± 0.24 *
PE61	0.002 ± 0.0001 *	0.69 ± 0.03 *	0.00053 ± 2.68 × 10^−5^ *	797.55 ± 39.87 *
PE85	3.65 ± 0.18 *	36.51 ± 1.82 *	0.38 ± 0.019	3360.34 ± 168.01 *
PE86	2.28 ± 0.11	0.28 ± 0.01 *	0.0018 ± 9.01 × 10^−5^ *	1617.93 ± 80.89 *

Data are presented as fold of increase respect to untreated cells, ± standard deviation. * *P* < 0.05 respect to lipopolysaccharide (LPS) treatment.

## Supplementary Materials

The following are available online at https://www.mdpi.com/2076-2607/7/8/254/s1, Table S1: PCR primers used in quantitative Real-Time PCR assay, Table S2: Antibiotic susceptibility measured by Etest method and expressed as percentage (%), Table S3: Virulence factors genes and antibiotic resistance related genes, Figure S1: Antagonistic activity of some LAB isolates against *Escherichia coli* ATCC 25922 (A and E panels), *Salmonella enterica* serovar *typhimurium* ATCC 14028 (panel B), *Staphylococcus aureus* ATCC 6538 (panel C), and *Listeria monocytogenes* DSM 12464 (panel D).
