# Supplementary material for: Piacentinu Ennese PDO Cheese as Reservoir of Promising Probiotic Bacteria
Source: Microorganisms. 2019 Aug 12;7(8):254. doi: 10.3390/microorganisms7080254 (PMC6723934; doi:10.3390/microorganisms7080254)
Supplement: Supplementary file 1 [file microorganisms-07-00254-s001.pdf]

Table S1. PCR primers used in quantitative Real-Time PCR assay

Gene	Forward Primer	Reverse Primer
COX-1	CTCCGGTTCTTGCTGTTCCT	GTCACACTGGTAGCGGTCAA
COX-2	CAAATTGCTGGCAGGGTTGC	AGGGCTTCAGCATAAAGCGT
GAPDH	AGACACCATGGGGAAGGTGA	TGGAATTTGCCATGGGTGGA
IL-8	TTTTGCCAAGGAGTGCTAAAGA	AACCCTCTGCACCCAGTTTTC
IL-10	GACTTTAAGGGTTACCTGGGTTG	TCACATGCGCCTTGATGTCTG

Table S2. Antibiotic susceptibility measured by Etest method and expressed as percentage (%)

Species	%	AMP	VA	GEN	KAN	STRE	ERY	CLIN	TC	CAP
L. plantarum (n=30)	R	0	nr	0	0	nr	0	0	0	0
	S	100	nr	100	100	nr	100	100	100	100
P. pentosaceus (n=27)	R	0	nr	0	0	18.5	0	0	0	0
	S	100	nr	100	100	81.5	100	100	100	100
L. rhamnosus (n=19)	R	0	nr	0	0	0	0	0	0	0
	S	100	nr	100	100	100	100	100	100	100
L. pentosus (n=17)	R	0	nr	0	0	nr	0	0	0	0
	S	100	nr	100	100	nr	100	100	100	100
L. paracasei (n=15)	R	0	nr	0	0	0	0	0	0	0
	S	100	nr	100	100	100	100	100	100	100
Leuc. lactis (n=4)	R	0	nr	0	50	0	0	0	0	0
	S	100	nr	100	50	100	100	100	100	100
% of resistance (R)		0	nr	0	1.8	4.5	0	0	0	0
% of susceptibility (S)		100	nr	100	98.2	95.5	100	100	100	100

Legend: R (resistant), S (susceptible), nr (not required) according to EFSA 2012.
AMP, ampicillin; VA, vancomycin; GEN, gentamycin; KAN, kanamycin; STRE, streptomycin; EY, erythromycin; CLIN, clindamycin; TC, tetracycline; CAP, chloramphenicol.

Table S3. Virulence factors genes and antibiotic resistance related genes.

Target genes	Tested strains					
	PE24	PE25	PE44	PE61	PE85	PE86
*gel*E	-	-	-	-	-	-
hyl	-	-	-	-	-	-
*asa*1	-	-	-	-	-	-
esp	-	-	-	-	-	-
*cyl*A	-	-	-	-	-	-
*efa*A	-	-	-	-	-	-
ace	-	-	-	-	-	-
ermA	-	-	-	-	-	-
ermB	-	-	-	-	-	-
ermC	-	-	-	-	-	-
tetK	-	-	-	-	-	-
tetL	-	-	-	-	-	-
tetM,	-	-	-	-	-	-
tetO	-	-	-	-	-	-
tetS	-	-	-	-	-	-
aac(6′)-Ie-aph(2″)-Ia	-	-	-	-	-	-
catA	-	-	-	-	-	-
aph(3′)-IIIa	-	-	-	-	-	-
ant(4′)-Ia	-	-	-	-	-	-
aph(2″)-Id	-	-	-	-	-	-
aph(2″)-Ic	-	-	-	-	-	-
aph(2″)-Ib	-	-	-	-	-	-
ant(6)-Ia	-	-	-	-	-	-

(+) presence, (-) absence of virulence factors genes and antibiotic resistance related genes.

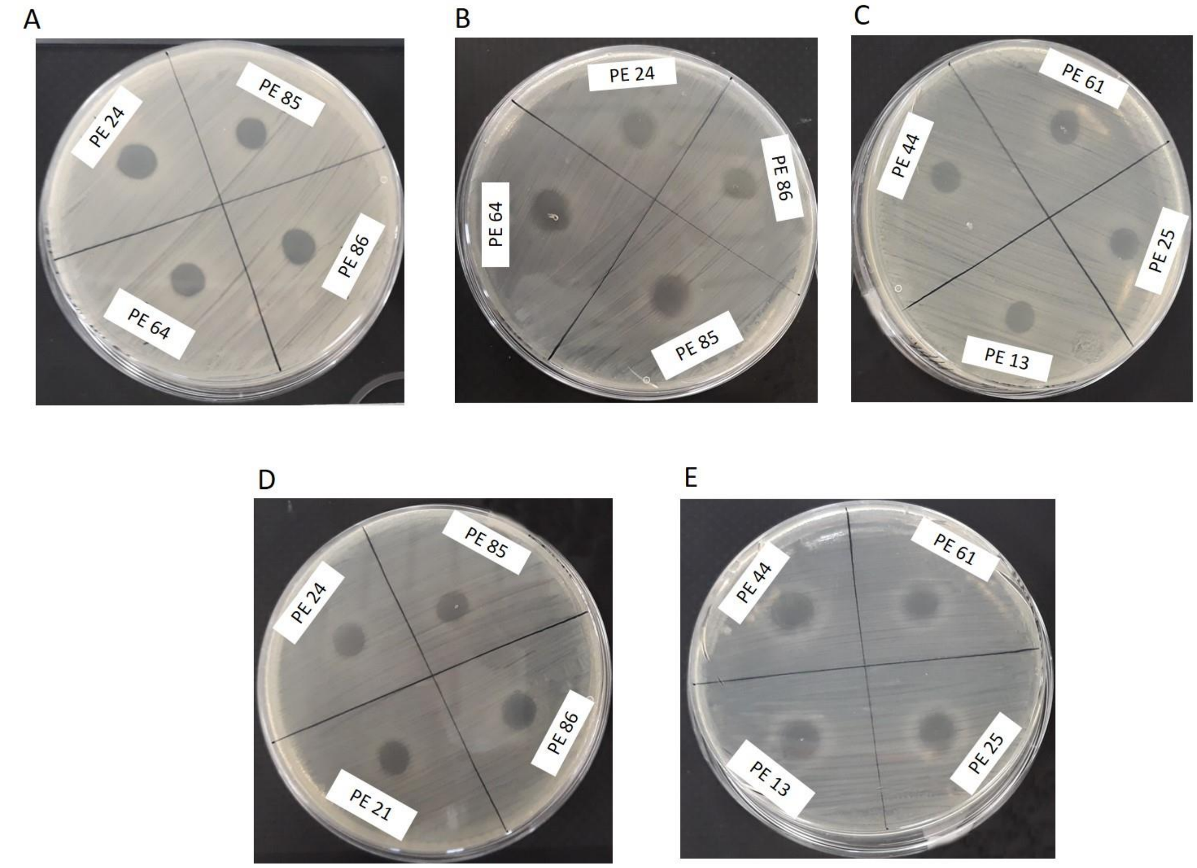

Figure S1. Antagonistic activity of some LAB isolates against *Escherichia coli* ATCC 25922 (A and E panels), *Salmonella enterica* serovar *typhimurium* ATCC 14028 (panel B), *Staphylococcus aureus* ATCC 6538 (panel C), and *Listeria monocytogenes* DSM 12464 (panel D).

© 2019 by the authors. Licensee MDPI, Basel, Switzerland. This article is an open access article distributed under the terms and conditions of the Creative Commons Attribution (CC BY) license (http://creativecommons.org/licenses/by/4.0/).
